# (*Z*)-2-(2-Chloro-3,3,3-trifluoro­prop-1-en­yl)-6-methoxy­phenyl acetate

**DOI:** 10.1107/S160053680801489X

**Published:** 2008-05-30

**Authors:** Hua Zhou, Zhi-Gang Li, Jing-Wei Xu

**Affiliations:** a National Analytical Research Center of Electrochemistry and Spectroscopy, Changchun Institute of Applied Chemistry, Chinese Academy of Sciences, Changchun 130022, People’s Republic of China, and, Graduate School of Chinese Academy of Sciences, Beijing 100039, People’s Republic of China

## Abstract

The crystal structure of the title compound, C_12_H_10_ClF_3_O_3_, was determined in order to establish the configuration of the C=double bond. The compound was found to be the *Z* isomer. The crystal structure is dominated by Cl⋯O halogen bonds [Cl⋯O = 3.111 (3) Å], as well as C—H⋯O and C—H⋯F hydrogen-bonding inter­actions, that connect neighboring mol­ecules into a three-dimensional supra­molecular network.

## Related literature

For related literature, see: Dmowski (1985 ▶); Fujita & Hiyama (1986 ▶); Nenajdenko *et al.*(2005 ▶); Politzer *et al.* (2007 ▶).
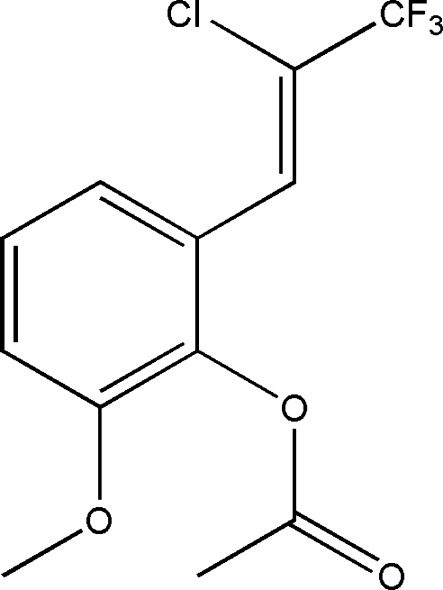


## Experimental

### 

#### Crystal data

C_12_H_10_ClF_3_O_3_
*M* *_r_* = 294.65
Triclinic, 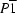

*a* = 8.6168 (19) Å
*b* = 8.6850 (19) Å
*c* = 9.723 (2) Å
α = 77.323 (3)°
β = 70.869 (3)°
γ = 84.010 (3)°
*V* = 670.3 (3) Å^3^
*Z* = 2
Mo *K*α radiation
μ = 0.32 mm^−1^
*T* = 293 (2) K
0.16 × 0.10 × 0.09 mm

#### Data collection

Bruker APEX CCD area-detector diffractometer
Absorption correction: multi-scan (*SADABS*; Sheldrick, 1996 ▶) *T*_min_ = 0.943, *T*_max_ = 0.968
3677 measured reflections
2551 independent reflections
1967 reflections with *I* > 2σ(*I*)
*R*_int_ = 0.009

#### Refinement

*R*[*F* ^2^ > 2σ(*F* ^2^)] = 0.052
*wR*(*F* ^2^) = 0.146
*S* = 1.03
2551 reflections
174 parameters
H-atom parameters constrained
Δρ_max_ = 0.31 e Å^−3^
Δρ_min_ = −0.20 e Å^−3^

### 

Data collection: *SMART* (Bruker, 1998 ▶); cell refinement: *SAINT-Plus* (Bruker, 2003 ▶); data reduction: *SAINT-Plus*; program(s) used to solve structure: *SHELXS97* (Sheldrick, 2008 ▶); program(s) used to refine structure: *SHELXL97* (Sheldrick, 2008 ▶); molecular graphics: *SHELXTL* (Sheldrick, 2008 ▶); software used to prepare material for publication: *SHELXTL*.

## Supplementary Material

Crystal structure: contains datablocks global, I. DOI: 10.1107/S160053680801489X/zl2108sup1.cif

Structure factors: contains datablocks I. DOI: 10.1107/S160053680801489X/zl2108Isup2.hkl

Additional supplementary materials:  crystallographic information; 3D view; checkCIF report

## Figures and Tables

**Table 1:** Hydrogen-bond geometry (Å, °)

*D*—H⋯*A*	*D*—H	H⋯*A*	*D*⋯*A*	*D*—H⋯*A*
C6—H6⋯F1^i^	0.93	2.64	3.508 (3)	156
C7—H7⋯O2^ii^	0.93	2.60	3.430 (3)	149

Symmetry codes: (i) 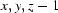; (ii) 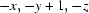.

**Fig. 1.**
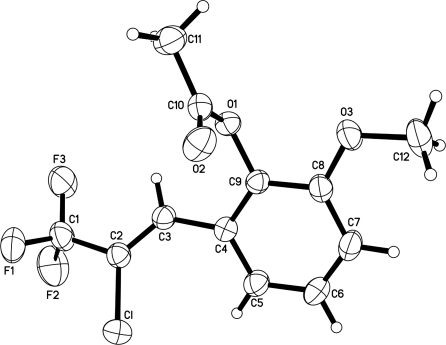
The structure of the title compound, showing the atomic numbering scheme. Displacement ellipsoids are drawn at the 30% probability level.

**Fig. 2.**
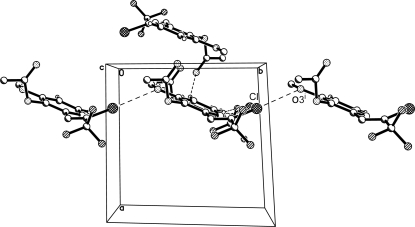
Perspective view of the packing structure of the title compound along the c axis. For the sake of clarity, H atoms not involved in the motifs shown have been omitted. Symmetry equivalent atoms marked i are created by the operator x, y+ 1, z).

## supplementary crystallographic information

### Comment

The title compound is an intermediate in the synthesis of trifluoromethyl substituted benzofurans. The configuration of analogue compounds such as 2-fluoro-3,3,3-trifluoroprop-1-enes (Dmowski, 1985), 2-chloro-3,3,3-trifluoroprop-1-enes (Fujita & Hiyama, 1986) or 2-bromo-3,3,3-trifluoroprop-1-enes (Nenajdenko *et al.*, 2005) were determined by ^1^H and ^19^F NMR. The configuration of the title compound, however, could not be determined with enough confidence by ^1^H and ^19^F NMR due to lack of data such as hetero-nuclear coupling constants, and its crystal structure was determined instead to determine its configuiration.

As shown in Fig. 1, the title compound is the *Z* isomer with the phenyl ring and the Cl atom on the same side of the C═C double bond. The C═C double bond and the ester bond have a large torsional angle with the phenyl ring with a tilting angle of the double bond and a dihedral angle between the planes of the ester and the phenyl ring of 66.01 (4)° and 83.15 (3)°, respectively. The ether bond, on the other hand, is nearly coplanar with the the phenyl ring, with a dihedral angle between the normal of the phenyl ring plane and the ether bond of 87.34 (3)°.

The molecular packing is stablized by Cl···O halogen bonds (Politzer *et al.*, 2007) between the Cl atom and the oxygen of a neighbouring ether bond, with a Cl—O3^i^ distance of 3.111 (3) Å (symmetry code as in Fig. 2) and a nearly linear C—Cl···O3^i^ angle of 178.0 (2)°. In addition, intermolecular C—H···O and C—H···F hydrogen bonds are present (Table 1 and Fig. 2). The two kinds of interactions lead to a three-dimensional supramolecular network. (Fig. 2).

### Experimental

The title compound was synthesized by a modified literature procedure (Fujita & Hiyama, 1986). Zinc powder (3.25 g, 50 mmol) and acetic anhydride (3.06 g, 30 mmol) were added into a solution of 2-hydroxy-3-methoxybenzaldehyde (1.52 g,10 mmol) in DMF (20 ml, dried by 4Å molecular sieve) under an argon atmosphere at room temperature. Then 1,1,1-trichloro-2,2,2-trifluoroethane (5.63 g, 30 mmol) was added dropwise to the mixture over ten minutes with fierce stirring. The reaction was monitored by thin layer chromatography. After completion, the reaction mixture was treated with saturated aqueous ammonium chloride solution (150 ml), and extracted with diethyl ether (3 × 50 ml). The organic phase was dried with magnesium sulfate, concentrated, and purification by silica gel column chromatography using petroleum ether as the eluent (R_f_ = 0.15). The purified product was recrystallized from petroleum ether to obtain colorless platelike crystals (1.47 g, 50%).

### Refinement

H atoms were placed geometrically and refined with fixed individual displacement parameters [*U*_iso_(H) = 1.2*U*_eq_(C,*N*)] (1.5 for methyl H atoms), using a riding model with C—H distances of 0.93 Å for C*sp*^2^ and 0.96 Å for methyl H atoms.

### Crystal data

**Table:**

C_12_H_10_ClF_3_O_3_	*Z* = 2
*M**_r_* = 294.65	*F*_000_ = 300
Triclinic, *P*1	*D*_x_ = 1.460 Mg m^−^^3^
Hall symbol: -P 1	Mo *K*α radiation λ = 0.71073 Å
*a* = 8.6168 (19) Å	Cell parameters from 1359 reflections
*b* = 8.6850 (19) Å	θ = 2.3–24.8º
*c* = 9.723 (2) Å	µ = 0.32 mm^−^^1^
α = 77.323 (3)º	*T* = 293 (2) K
β = 70.869 (3)º	Sheet, colorless
γ = 84.010 (3)º	0.16 × 0.10 × 0.09 mm
*V* = 670.3 (3) Å^3^	

### Data collection

**Table:**

Bruker APEX CCD area-detector diffractometer	2551 independent reflections
Radiation source: fine-focus sealed tube	1967 reflections with *I* > 2σ(*I*)
Monochromator: graphite	*R*_int_ = 0.009
*T* = 293(2) K	θ_max_ = 26.0º
φ and ω scans	θ_min_ = 2.3º
Absorption correction: multi-scan(SADABS; Sheldrick, 1996)	*h* = −10→10
*T*_min_ = 0.943, *T*_max_ = 0.968	*k* = −10→4
3677 measured reflections	*l* = −11→11

### Refinement

**Table:**

Refinement on *F*^2^	Secondary atom site location: difference Fourier map
Least-squares matrix: full	Hydrogen site location: inferred from neighbouring sites
*R*[*F*^2^ > 2σ(*F*^2^)] = 0.052	H-atom parameters constrained
*wR*(*F*^2^) = 0.146	*w* = 1/[σ^2^(*F*_o_^2^) + (0.0746*P*)^2^ + 0.2352*P*] where *P* = (*F*_o_^2^ + 2*F*_c_^2^)/3
*S* = 1.03	(Δ/σ)_max_ = 0.026
2551 reflections	Δρ_max_ = 0.31 e Å^−^^3^
174 parameters	Δρ_min_ = −0.19 e Å^−^^3^
Primary atom site location: structure-invariant direct methods	Extinction correction: none

### Special details

**Table:**

Geometry. All e.s.d.'s (except the e.s.d. in the dihedral angle between two l.s. planes) are estimated using the full covariance matrix. The cell e.s.d.'s are taken into account individually in the estimation of e.s.d.'s in distances, angles and torsion angles; correlations between e.s.d.'s in cell parameters are only used when they are defined by crystal symmetry. An approximate (isotropic) treatment of cell e.s.d.'s is used for estimating e.s.d.'s involving l.s. planes.
Refinement. Refinement of *F*^2^ against ALL reflections. The weighted *R*-factor *wR* and goodness of fit *S* are based on *F*^2^, conventional *R*-factors *R* are based on *F*, with *F* set to zero for negative *F*^2^. The threshold expression of *F*^2^ > σ(*F*^2^) is used only for calculating *R*-factors(gt) *etc*. and is not relevant to the choice of reflections for refinement. *R*-factors based on *F*^2^ are statistically about twice as large as those based on *F*, and *R*- factors based on ALL data will be even larger.

### Fractional atomic coordinates and isotropic or equivalent isotropic displacement parameters (Å^2^)

**Table:**

	*x*	*y*	*z*	*U*_iso_*/*U*_eq_	
Cl	0.29665 (10)	0.96976 (8)	0.19741 (8)	0.0749 (3)	
F1	0.3028 (3)	0.8620 (2)	0.5096 (2)	0.0953 (6)	
F2	0.5445 (3)	0.9077 (3)	0.3604 (3)	0.1170 (8)	
F3	0.4779 (3)	0.6737 (3)	0.4667 (2)	0.1112 (8)	
O1	0.24346 (19)	0.36598 (19)	0.26609 (17)	0.0504 (4)	
O2	−0.0223 (2)	0.4383 (3)	0.3538 (2)	0.0725 (6)	
O3	0.1566 (2)	0.2860 (2)	0.0539 (2)	0.0692 (5)	
C1	0.4235 (4)	0.8063 (4)	0.4042 (4)	0.0719 (8)	
C2	0.3680 (3)	0.7914 (3)	0.2776 (3)	0.0538 (6)	
C3	0.3712 (3)	0.6540 (3)	0.2383 (3)	0.0508 (6)	
H3	0.4071	0.5672	0.2966	0.061*	
C4	0.3255 (3)	0.6202 (3)	0.1150 (3)	0.0481 (5)	
C5	0.3518 (3)	0.7237 (3)	−0.0219 (3)	0.0602 (7)	
H5	0.3972	0.8211	−0.0378	0.072*	
C6	0.3106 (4)	0.6812 (4)	−0.1326 (3)	0.0674 (7)	
H6	0.3287	0.7509	−0.2234	0.081*	
C7	0.2429 (3)	0.5379 (4)	−0.1129 (3)	0.0630 (7)	
H7	0.2137	0.5126	−0.1890	0.076*	
C8	0.2185 (3)	0.4316 (3)	0.0209 (3)	0.0539 (6)	
C9	0.2602 (3)	0.4751 (3)	0.1335 (2)	0.0467 (5)	
C10	0.0917 (3)	0.3562 (3)	0.3710 (3)	0.0519 (6)	
C11	0.0957 (4)	0.2335 (4)	0.5034 (3)	0.0744 (8)	
H11A	0.0105	0.2571	0.5893	0.112*	
H11B	0.2006	0.2323	0.5184	0.112*	
H11C	0.0788	0.1320	0.4878	0.112*	
C12	0.1195 (4)	0.2342 (4)	−0.0610 (4)	0.0830 (9)	
H12A	0.0436	0.3086	−0.0959	0.124*	
H12B	0.0713	0.1326	−0.0228	0.124*	
H12C	0.2188	0.2266	−0.1416	0.124*	

### Atomic displacement parameters (Å^2^)

**Table:**

	*U*^11^	*U*^22^	*U*^33^	*U*^12^	*U*^13^	*U*^23^
Cl	0.1011 (6)	0.0514 (4)	0.0688 (5)	0.0024 (4)	−0.0266 (4)	−0.0073 (3)
F1	0.1230 (16)	0.1053 (15)	0.0688 (11)	0.0132 (12)	−0.0362 (11)	−0.0389 (11)
F2	0.1136 (16)	0.1319 (19)	0.1389 (19)	−0.0336 (14)	−0.0565 (14)	−0.0555 (16)
F3	0.173 (2)	0.0917 (14)	0.1183 (16)	0.0367 (14)	−0.1100 (16)	−0.0424 (12)
O1	0.0533 (9)	0.0474 (9)	0.0512 (9)	0.0015 (7)	−0.0205 (8)	−0.0064 (7)
O2	0.0595 (11)	0.0929 (15)	0.0630 (12)	0.0129 (10)	−0.0226 (9)	−0.0131 (10)
O3	0.0895 (14)	0.0579 (11)	0.0799 (13)	−0.0021 (10)	−0.0460 (11)	−0.0243 (10)
C1	0.085 (2)	0.0677 (18)	0.080 (2)	0.0004 (16)	−0.0379 (17)	−0.0311 (16)
C2	0.0559 (14)	0.0541 (14)	0.0538 (14)	−0.0044 (11)	−0.0185 (11)	−0.0126 (11)
C3	0.0541 (13)	0.0516 (13)	0.0490 (13)	−0.0053 (11)	−0.0194 (11)	−0.0081 (11)
C4	0.0474 (12)	0.0524 (13)	0.0452 (13)	−0.0021 (10)	−0.0151 (10)	−0.0100 (10)
C5	0.0684 (16)	0.0609 (16)	0.0483 (14)	−0.0125 (13)	−0.0152 (12)	−0.0050 (12)
C6	0.0817 (19)	0.0748 (19)	0.0430 (14)	−0.0036 (15)	−0.0201 (13)	−0.0044 (13)
C7	0.0689 (17)	0.0790 (19)	0.0505 (15)	0.0093 (14)	−0.0290 (13)	−0.0219 (13)
C8	0.0564 (14)	0.0557 (15)	0.0586 (15)	0.0065 (11)	−0.0259 (12)	−0.0217 (12)
C9	0.0474 (12)	0.0484 (13)	0.0452 (12)	0.0042 (10)	−0.0177 (10)	−0.0092 (10)
C10	0.0590 (15)	0.0514 (13)	0.0510 (14)	−0.0020 (12)	−0.0214 (11)	−0.0151 (11)
C11	0.0800 (19)	0.0706 (19)	0.0617 (17)	−0.0029 (15)	−0.0159 (15)	−0.0005 (14)
C12	0.091 (2)	0.084 (2)	0.098 (2)	0.0006 (18)	−0.0437 (19)	−0.0489 (19)

### Geometric parameters (Å, °)

**Table:**

Cl—C2	1.724 (3)	C5—C6	1.368 (4)
F1—C1	1.334 (4)	C5—H5	0.9300
F2—C1	1.335 (4)	C6—C7	1.378 (4)
F3—C1	1.297 (4)	C6—H6	0.9300
O1—C10	1.367 (3)	C7—C8	1.386 (4)
O1—C9	1.397 (3)	C7—H7	0.9300
O2—C10	1.188 (3)	C8—C9	1.392 (3)
O3—C8	1.356 (3)	C10—C11	1.488 (4)
O3—C12	1.427 (3)	C11—H11A	0.9600
C1—C2	1.493 (4)	C11—H11B	0.9600
C2—C3	1.325 (3)	C11—H11C	0.9600
C3—C4	1.472 (3)	C12—H12A	0.9600
C3—H3	0.9300	C12—H12B	0.9600
C4—C9	1.385 (3)	C12—H12C	0.9600
C4—C5	1.397 (3)		
			
C10—O1—C9	117.36 (18)	C6—C7—H7	120.1
C8—O3—C12	117.3 (2)	C8—C7—H7	120.1
F3—C1—F1	107.4 (3)	O3—C8—C7	125.8 (2)
F3—C1—F2	106.7 (3)	O3—C8—C9	115.7 (2)
F1—C1—F2	106.0 (2)	C7—C8—C9	118.5 (2)
F3—C1—C2	113.2 (2)	C4—C9—C8	121.9 (2)
F1—C1—C2	111.7 (3)	C4—C9—O1	119.1 (2)
F2—C1—C2	111.4 (3)	C8—C9—O1	118.9 (2)
C3—C2—C1	122.2 (2)	O2—C10—O1	122.5 (2)
C3—C2—Cl	126.0 (2)	O2—C10—C11	127.4 (3)
C1—C2—Cl	111.8 (2)	O1—C10—C11	110.1 (2)
C2—C3—C4	128.8 (2)	C10—C11—H11A	109.5
C2—C3—H3	115.6	C10—C11—H11B	109.5
C4—C3—H3	115.6	H11A—C11—H11B	109.5
C9—C4—C5	118.3 (2)	C10—C11—H11C	109.5
C9—C4—C3	118.2 (2)	H11A—C11—H11C	109.5
C5—C4—C3	123.4 (2)	H11B—C11—H11C	109.5
C6—C5—C4	119.8 (3)	O3—C12—H12A	109.5
C6—C5—H5	120.1	O3—C12—H12B	109.5
C4—C5—H5	120.1	H12A—C12—H12B	109.5
C5—C6—C7	121.7 (3)	O3—C12—H12C	109.5
C5—C6—H6	119.2	H12A—C12—H12C	109.5
C7—C6—H6	119.2	H12B—C12—H12C	109.5
C6—C7—C8	119.7 (2)		

### Hydrogen-bond geometry (Å, °)

**Table:**

*D*—H···*A*	*D*—H	H···*A*	*D*···*A*	*D*—H···*A*
C6—H6···F1^i^	0.93	2.64	3.508 (3)	156
C7—H7···O2^ii^	0.93	2.60	3.430 (3)	149

Symmetry codes: (i) *x*, *y*, *z*−1; (ii) −*x*, −*y*+1, −*z*.
